# Supplementary material for: Beta-Hydroxybutyrate Augments Oxaliplatin-Induced Cytotoxicity by Altering Energy Metabolism in Colorectal Cancer Organoids
Source: Cancers (Basel). 2023 Dec 6;15(24):5724. doi: 10.3390/cancers15245724 (PMC10741617; doi:10.3390/cancers15245724)
Supplement: Supplementary file 1 [file cancers-15-05724-s001.zip › cancers-2638589-supplementary.pdf]

Supplementary Materials

Beta-Hydroxybutyrate Augments Oxaliplatin-Induced Cytotoxicity by Altering Energy Metabolism in Colorectal Cancer Organoids

Tolga Sever [1], Ender Berat Ellidokuz [2], Yasemin Basbinar [3], Hulya Ellidokuz [4], Ömer H. Yilmaz [5,6,7] and Gizem Calibasi-Kocal [8,9]

1 Department of Translational Oncology, Institute of Health Sciences, Dokuz Eylul University, 35340 Izmir, Turkey
2 Department of Internal Diseases, Gastroenterology, Faculty of Medicine, Dokuz Eylul University, 35340 Izmir, Turkey
3 Department of Translational Oncology, Institute of Oncology, Dokuz Eylul University, 35340 Izmir, Turkey
4 Department of Preventive Oncology, Institute of Oncology, Dokuz Eylul University, 35340 Izmir, Turkey
5 Department of Biology, The David H. Koch Institute for Integrative Cancer Research at MIT, Massachusetts Institute of Technology, Cambridge, MA 02139, USA
6 Broad Institute of MIT and Harvard, Cambridge, MA 02142, USA
7 Department of Pathology, Massachusetts General Hospital and Harvard Medical School, Boston, MA 02114, USA
8 Department of Translational Oncology, Institute of Oncology, Dokuz Eylul University, 35340 Izmir, Turkey
9 Koch Institute for Integrative Cancer Research at MIT, Cambridge, MA 02139, USA
* Correspondence: gizem.calibasi@deu.edu.tr

Supplementary Method

1. Optimization of organoid culture for drug screening

The organoid generation capacities of healthy colon and CRC organoids for 5 days under optimal growth mediums. As shown in Figure 1, organoids reached their maximum capacity on day 4 and after that, they started to create necrotic areas. Thus, we indicated that 96 hours of cultivation should be used upon treatment applications.

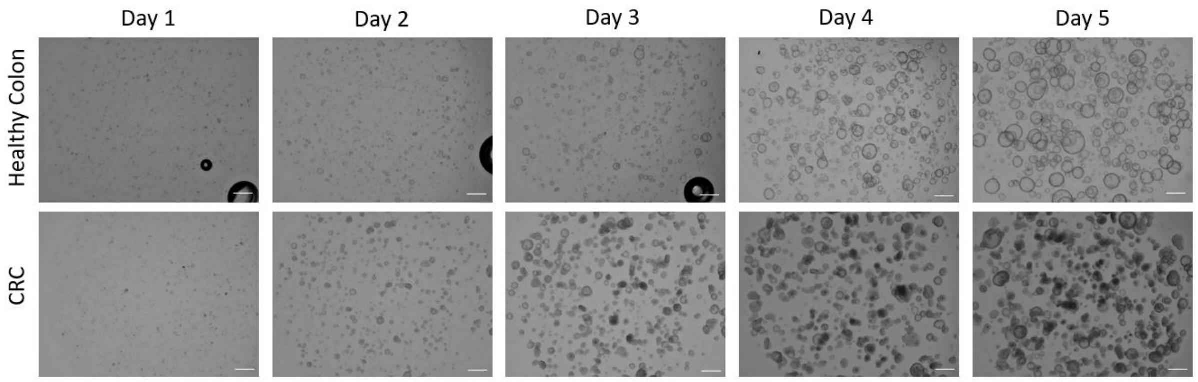

Supplementary Figure S1. Organoid generation capacity. To evaluate the maximum generation capacity, 10^3 single cells were derived from a healthy colon and CRC organoids were cultured in 48-well plates. Images were taken daily for 5 days by Confocal LSM 880, Zeiss at 5x magnitude. The scale bar is 200μm.

Supplementary Results

1. Western Blot Analysis

Treatments consisting of 10mM β-hydroxybutyrate (BOHB), 200μM melatonin, and a range of oxaliplatin (OXA) concentrations between IC10 and IC50 were applied to colorectal cancer organoids cultured in 48-well plates for a duration of 96 hours. The samples due to the treatments and their corresponding combinations, loaded sequentially onto the gel as follows: 1. Melatonin+IC10 OXA, 2. Melatonin+IC50 OXA, 3. Melatonin, 4. BOHB, 5. Melatonin+BOHB, 6. Melatonin+BOHB+IC50 OXA, 7. Melatonin+BOHB+IC10 OXA, 8. BOHB+IC50 OXA, 9. BOHB+IC10 OXA, 10. IC10 Oxa 11.IC50 Oxa, 12. Control. Western blot images with respective densitometry data and leader aligned with the experimental design were illustrated in Supplementary Figures 2, 3, and 4 for different gels.

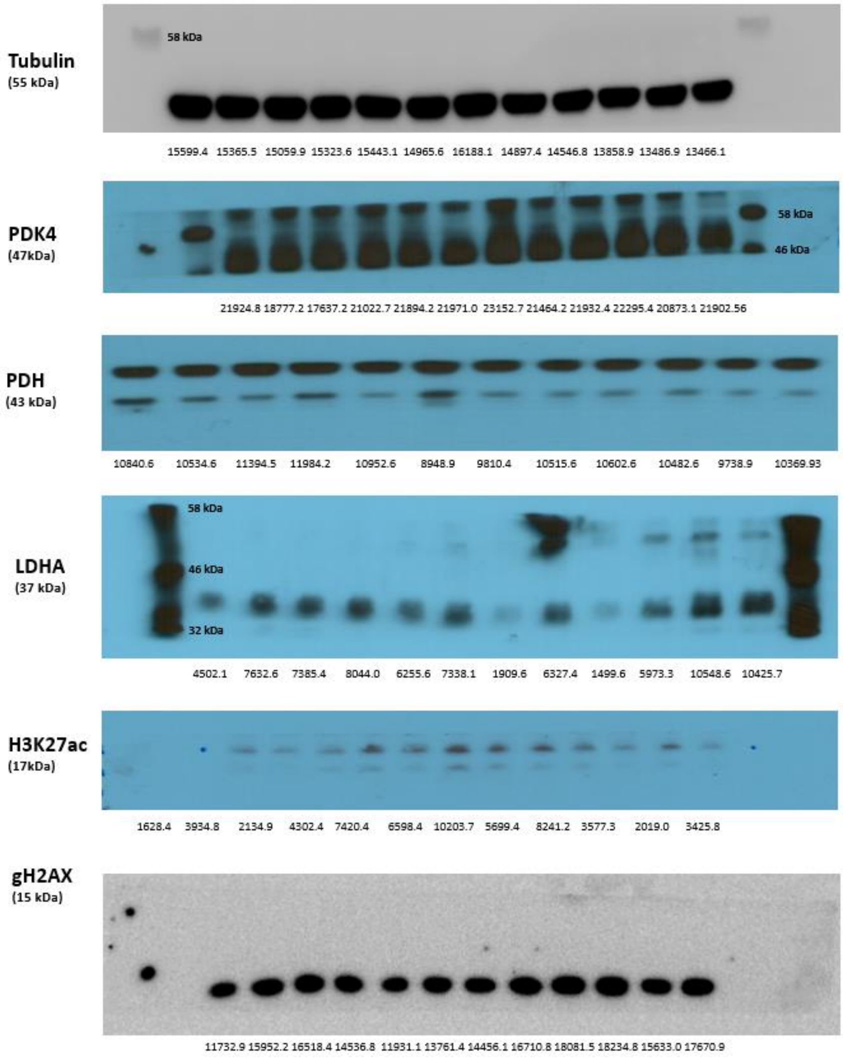

Supplementary Figure S2. The western blot images for for the following proteins in order of their molecular weights: Tubulin, pyruvate dehydrogenase lipoamide kinase isozyme 4 (PDK4), pyruvate dehydrogenase (PDH), lactate dehydrogenase A (LDHA), Histone H3K27 acetylation (H3K27ac), and Gamma-H2AX (gH2AX). The individual densitometry data corresponding to each protein band was placed below the respective bands.

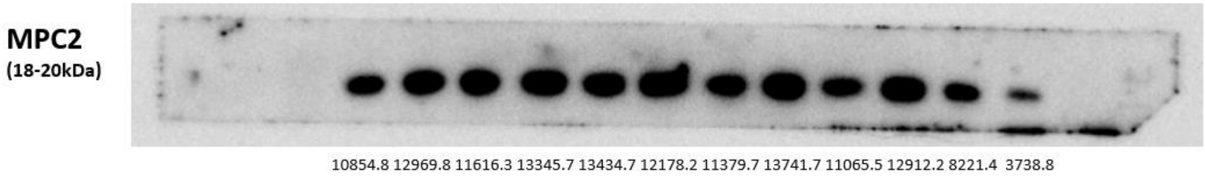

Supplementary Figure S3. A Western blot raw image for the protein mitochondrial pyruvate carrier-2 (MPC2) was arranged in order of molecular weight. Individual densitometry data was inserted below the bands, ensuring proper presentation and scientific accuracy.

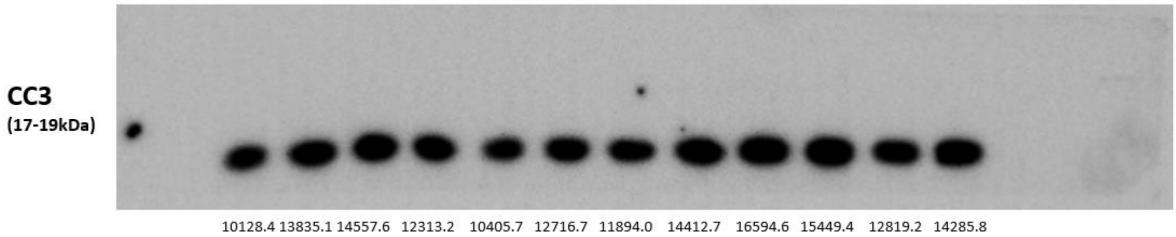

Supplementary Figure S4. A Western blot raw image for the protein cleaved caspase-3 (CC3) was arranged in order of molecular weight. Individual densitometry data was inserted below the bands, ensuring proper presentation and scientific accuracy.

2. BOHB does not enhance oxaliplatin cytotoxicity effect on healthy colon organoids

Oxaliplatin was administered on healthy colon organoids and the IC10 value was detected as 89 nm. As shown in Figure 5, combinations of BOHB and Oxaliplatin IC10 doses were applied to healthy colon organoids, but BOHB-enhanced Oxaliplatin cytotoxicity could not be observed.

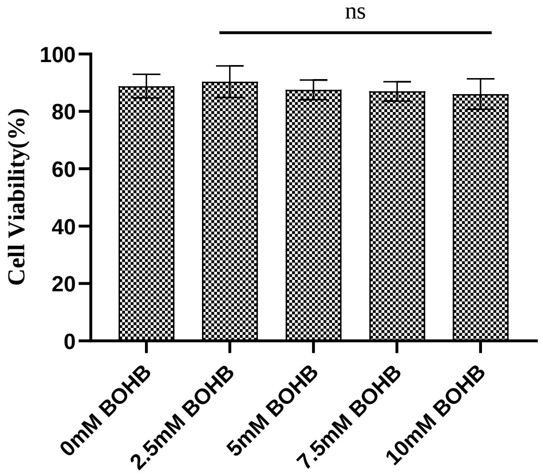

Supplementary Figure S5. Assessment of Cytotoxic Effects of BOHB, and Oxaliplatin on Normal Colon Organoids. Single cells derived from normal colon organoids were cultured in 48-well plates and subjected to IC10 value of Oxaliplatin and 0-2.5-5.7.5-10mM BOHB. Following 96 hours of treatment, cell viability was evaluated using a resazurin assay. All experiments were performed in fourfold replication, both technically and biologically. Cytotoxic impacts of the treatments are demonstrated as percentages relative to the untreated controls (100%). Asterisks indicate treatments with significant cytotoxic effects ($p < 0.05$, compared to the control).
